# Well‐Being, Inflammation, and Physical Activity in Acute and Chronic Back Pain: A Cross‐Sectional Analysis of 22,864 UK Biobank Participants

**DOI:** 10.1002/ejp.70079

**Published:** 2025-07-20

**Authors:** Romina Gollan, Patrick J. Owen, Jamie L. Tait, Luana C. Main

**Affiliations:** ^1^ Institute for Physical Activity and Nutrition (IPAN), School of Exercise and Nutrition Sciences Deakin University Geelong Victoria Australia; ^2^ Eastern Health Emergency Medicine Program Melbourne Victoria Australia; ^3^ Eastern Health Clinical School Monash University Melbourne Victoria Australia

## Abstract

**Background:**

Back pain is influenced by biological, psychological, and social factors, yet is often investigated separately.

**Methods:**

Cross‐sectional analysis of the UK Biobank comparing well‐being (Patient Health Questionnaire‐4; 4–16 points; higher scores indicate greater levels of depressive and anxiety symptoms), number of stressful life events, C‐reactive protein (CRP), and physical activity (International Physical Activity Questionnaire) among pain‐free, acute, and chronic back pain individuals. The sample included 22,864 individuals: 5716 with acute back pain, 5716 with chronic back pain, and 11,432 pain‐free controls. Group comparisons were performed using network analysis and analysis of covariance, adjusted for socioeconomic deprivation, body mass index, smoking, and alcohol consumption.

**Results:**

Well‐being was poorer in acute (mean difference [95% CI]: 0.20 [0.14, 0.25] points; *p* < 0.001) and chronic back pain (0.39 [0.34, 0.45] points; *p* < 0.001) compared to controls. More stressful life events were measured in acute (0.03 [0.01, 0.05] points; *p* = 0.041) and chronic back pain (0.03 [0.01, 0.05] points; *p* = 0.028) compared to controls. However, this finding was not robust to sensitivity analyses. Elevated CRP was found in acute (2.28 [0.57, 3.99]%; *p* = 0.024; ES = very small), but not in chronic back pain, compared to controls. No significant group differences were observed for physical activity. Network structures did not differ between groups.

**Conclusions:**

Differences in well‐being and CRP among pain‐free, acute, and chronic back pain individuals were identified, suggesting that variables may be affected by back pain temporality. Further prospective research incorporating additional variables is needed to explore the drivers of back pain.

**Significance Statement:**

Individuals with acute back pain showed 2% higher CRP than controls, though values remained within the normal range (< 5 mg/L) across groups and below clinical relevance, suggesting limited utility of CRP in distinguishing acute from chronic back pain. Poorer well‐being in both acute and chronic back pain underscores the need to explore underlying causal pathways. Moreover, similar network structures among groups indicate no neuroimmune involvement in back pain onset or chronification based on the variables examined.

## 1 Introduction

Back pain is influenced by biological, psychological, and social factors, as highlighted in the biopsychosocial model (Hartvigsen et al. 2018). Approximately one‐third of acute back pain (ABP; pain < 12 weeks) cases transition to chronic back pain (CBP; pain ≥ 12 weeks), leading to long‐term disability, poor well‐being (encompassing mental and social health), and increased healthcare costs (Driscoll et al. 2014; Stevans et al. 2021). Therefore, identifying factors contributing to ABP and CBP is crucial for improving treatment.

The neuroimmune system, involving interactions between the central nervous system (CNS) and immune system, is a proposed mechanism in chronic pain development (Hore and Denk 2019). Specifically, pro‐inflammatory cytokines may drive central sensitisation in CBP (Sanzarello et al. 2016). However, its role in back pain remains unexplored, with well‐being and inflammation largely studied separately. For example, one study reported associations between back pain and depressive episodes (odds ratio [OR] = 2.88), anxiety (OR = 2.12), and stress (OR = 1.13), with more pronounced associations for CBP (Stubbs et al. 2016). Regarding inflammation, systematic reviews suggested pro‐inflammatory cytokines, including C‐reactive protein (CRP), tumour necrosis factor‐alpha (TNF‐α), interleukin (IL)‐1β, and IL‐6, may contribute to back pain (Morris et al. 2020; Pinto et al. 2023). Yet, findings remain inconclusive due to small samples and limited differentiation between ABP and CBP. For example, the only systematic review assessing CRP while differentiating ABP from CBP included only two studies, one study on ABP (*n* = 99) as well as one on ABP and CBP (*n* = 72), finding elevated CRP levels in ABP, but no association in CBP (Morris et al. 2020). Further research is needed to clarify the role of inflammatory markers in pain chronification and its utility as clinical biomarkers.

Physical activity and exercise are first‐line recommendations for CBP (Alperovitch‐Najenson et al. 2023), offering neuroimmune‐modifying effects that reduce inflammation and provide neuroprotection in various chronic conditions (Archer et al. 2011; Spielman et al. 2016). However, the impact on the neuroimmune system in back pain remains unclear, as studies have only examined effects on the CNS and immune system separately (Kim et al. 2008; Owen et al. 2020). Understanding how physical activity interacts with well‐being and inflammation may clarify ABP‐to‐CBP progression.

Despite the biopsychosocial framework, research continues to examine factors separately, often with small samples (Otero‐Ketterer et al. 2022). In 214 studies, those with biological measures reported a mean sample of 238, compared to 960 for psychosocial measures (Yim et al. 2015). Consequently, the interrelationships between well‐being, inflammation, and physical activity remain poorly understood. To address these gaps, we used UK Biobank data to conduct the largest cross‐sectional and first network analysis of well‐being, stressful life events, inflammation, and physical activity in ABP and CBP. Network analysis examines interactions among multiple variables, providing a more comprehensive view than bivariate approaches (Borsboom et al. 2021). We aimed to (1) explore differences in these variables across ABP, CBP, and pain‐free individuals within the same cohort; and (2) examine bivariate and multivariate relationships between variables. This approach aims to clarify biopsychosocial interactions in back pain and to inform more targeted treatments.

## 2 Methods

### 2.1 Study Design and Data

**FIGURE 1:**
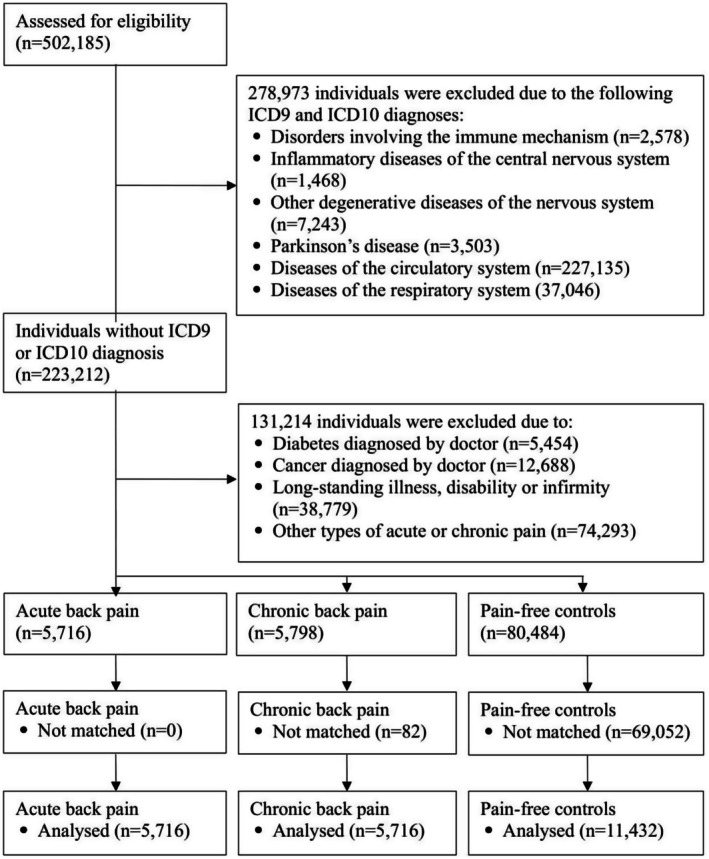
Flowchart of participant selection.

A population‐based, cross‐sectional study of 502,185 adults from the UK Biobank was conducted. The UK Biobank is a large‐scale database that comprises phenotypic and genetic data. Briefly, participants were recruited for the baseline measurements, with assessments conducted between 2006 and 2010 at 22 assessment centres across the United Kingdom. Data collected during this initial assessment included sociodemographic information, lifestyle factors, clinical diagnoses, physical measures, and biological samples. Detailed information on the UK Biobank project can be found elsewhere (Sudlow et al. 2015). The UK Biobank was approved by the NHS National Research Ethics Service (Ref 11/NW/0382) and the Deakin University Human Research Ethics Committee granted ethical exemption for this study (project ID: 2023–348). All participants provided written informed consent. For this study, only data from the baseline assessment (March 2006–December 2010) were used. Participants with “disorders involving the immune mechanism”; “inflammatory diseases of the CNS”; “other degenerative neurological diseases”; “Parkinson's disease”; “circulatory diseases”; or “respiratory diseases” (all diagnosed according to ICD‐9 or ICD‐10), “diabetes”; “cancer”; “other long‐standing illness, disability, or infirmity”; or other types of acute or chronic pain (e.g., headache, neck or shoulder pain) were excluded (Figure 1). The remaining participants were categorised into three exposure groups: (1) ABP (*n* = 5716), (2) CBP (*n* = 5798), and (3) pain‐free controls (*n* = 80,484). To control for potential confounding effects of age and sex and to enhance comparability, individuals were matched pairwise by age and sex through random selection of cases and controls (Fletcher et al. 2012). A matching ratio of 1:1:2 was applied, with individuals experiencing ABP matched to those with CBP and pain‐free controls. After matching, the groups were assessed for differences in well‐being, stressful life events, inflammation, and physical activity. The study adhered to the Strengthening the Reporting of Observational Studies in Epidemiology (STROBE) guidelines (Von Elm et al. 2007).

### 2.2 Exposure

Males and females (40–69 years of age) with ABP, CBP, and pain‐free controls were examined. Back pain status was self‐reported via a touchscreen questionnaire at baseline. Individuals were allocated to: (1) ABP if answering “yes” to back pain in the last month and “no” to have had back pain for more than 3 months; (2) CBP if answering “yes” to back pain in the last month and “yes” to have had back pain for more than 3 months; and (3) pain‐free controls if answering “none of the above” to the pain experienced in the last month. Participants who reported additional types of acute or chronic pain (e.g., headache, neck or shoulder pain) were excluded. This classification follows the standard definitions of ABP and CBP (Furlan et al. 2015).

### 2.3 Outcome Measures

Well‐being was measured using the 4‐item Patient Health Questionnaire (PHQ‐4), which comprises the 2‐item depression scale (PHQ‐2) and the 2‐item Generalised Anxiety Disorder scale (GAD‐2) (Kroenke et al. 2009; Lowe et al. 2010). The four questions covered the past 2 weeks and assessed (1) depressed mood (“How often have you felt down, depressed or hopeless?”); (2) disinterest or unenthusiasm (“How often have you had little interest or pleasure in doing things?”); (3) tenseness or restlessness (“How often have you felt tense, fidgety or restless?”); and (4) tiredness or lethargy (“How often have you felt tired or had little energy?”). Each item was scored on a 4‐point Likert scale (1 = not at all to 4 = nearly every day), with higher scores indicating poorer well‐being. This procedure aligns with previous UK Biobank studies (Dregan et al. 2020; Tagliaferri et al. 2022). The PHQ‐4 has demonstrated acceptable reliability (*α* = 0.77–0.88) and validity (*r* = 0.46–0.83) in the general population (Caro‐Fuentes and Sanabria‐Mazo 2023; Kroenke et al. 2009; Lowe et al. 2010).

Stressful life events were measured via a self‐reported touchscreen questionnaire asking participants if they had experienced any of the following in the past 2 years: (1) “serious illness, injury, or assault to yourself”; (2) “serious illness, injury, or assault of a close relative”; (3) “death of a close relative”; (4) “death of a spouse/partner”; (5) “marital separation/divorce”; or (6) “financial difficulties”. Participants were able to select multiple answers. Reported events were summed, ranging from 0 (none) to 6 (all).

Serum high‐sensitivity CRP as a major inflammatory biomarker was assessed by a 40–50 mL blood sample, which was collected and analysed during the UK Biobank baseline assessment. The analysis was limited to CRP as no further measures of inflammation were available within the UK Biobank. CRP was measured by the immunoturbidimetric method on a Beckman Coulter AU5800 chemistry analyser (Beckman Coulter Inc., Brea, United States of America) (Fry et al. 2019). CRP is expressed in milligrams per litre (mg/L). CRP was log‐transformed due to non‐normal distribution.

Physical activity was measured by the International Physical Activity Questionnaire (IPAQ) short form via the UK Biobank touchscreen questionnaire. Six questions regarding the duration and frequency of distinct levels of activity (walking, moderate, and vigorous physical activity) over the previous week were asked: (1) “In a typical week, on how many days did you walk for at least 10 min at a time?”; (2) “How many minutes did you usually spend walking on a typical day?”; (3) “In a typical week, on how many days did you do 10 min or more of moderate physical activities like carrying light loads, cycling at normal pace?”; (4) “How many minutes did you usually spend doing moderate activities on a typical day?”; (5) “In a typical week, how many days did you do 10 min or more of vigorous physical activity?”; (6) “How many minutes did you usually spend doing vigorous activities on a typical day?” For each activity, the number of days was multiplied by the number of minutes to estimate the total metabolic equivalent of task (MET) in minutes per week (min/week). Following IPAQ guidelines, values in which the sum of walking, moderate, and vigorous time exceeded 960 min were excluded as outliers (IPAQ Research Committee 2005). The IPAQ demonstrated acceptable reliability (*ρ* = 0.80) and validity (*ρ* = 0.30) among adults (Craig et al. 2003).

### 2.4 Covariates

Covariates included sex (female/male), age (years), socioeconomic deprivation, body mass index (BMI; kg/m^2^), smoking status (never, former, current), and alcohol consumption (never, special occasions only, 1–3 times per month, 1–2 times per week, 3–4 times per week, daily or almost daily) as these factors are associated with the outcome measures. Data on sex, age, and socioeconomic deprivation were obtained from the central registry at recruitment. Socioeconomic deprivation was assessed using the Townsend Deprivation Index, which combines four census‐based variables (households without a car, overcrowded households, non‐owner‐occupied households, and unemployment). Positive values indicate high material deprivation, whereas negative values indicate relative affluence, with zero representing mean scores (Townsend et al. 2023). BMI was calculated from height and weight measurements, and smoking status and alcohol consumption were self‐reported.

### 2.5 Statistical Analyses

Statistical analyses were conducted using RStudio Pro (version 2024.4.2.764.1; www.posit.co/) (Posit Team 2024). The R code is publicly available (https://github.com/RomyGollan/UKBiobank_back_pain.git) for reproducibility. The back pain and pain‐free control groups were regarded as independent variables, whereas the outcome measures (well‐being, stressful life events, inflammation, and physical activity) were considered as dependent variables. All outcome measures were treated as continuous data.

Initially, data were summarised using descriptive statistics (presented as mean (SD) or number (percentage) for continuous and categorical outcomes, respectively). One‐way analysis of variance (ANOVA) was then conducted, with residuals checked for normality. If residuals were not normally distributed, data were log‐transformed. Next, analysis of covariance (ANCOVA) was performed to evaluate differences between groups on each outcome, adjusting for covariates (socioeconomic deprivation, BMI, smoking status, and alcohol consumption). Post hoc pairwise comparisons calculated adjusted estimated marginal means and standard errors, using Tukey's honest significant difference to control for type I error across multiple comparisons. To assess the magnitude of differences among groups, confidence intervals and effect sizes (Cohen's *d*) were calculated, with effect sizes classified as very small (< 0.2), small (0.2–0.49), moderate (0.5–0.79), and large (> 0.8) (Cohen 1992).

To further investigate the relationship between dependent variables, adjusted partial correlation analyses were conducted across all groups and within each subgroup (ABP, CBP, and pain‐free controls), adjusting for age, socioeconomic deprivation, smoking status, alcohol consumption, BMI, and the other dependent variables not being analysed. The Benjamini‐Hochberg procedure was applied to control for false discovery rate, adjusting the *p*‐values derived from multiple hypothesis testing (Benjamini and Hochberg 1995). Correlation coefficients were interpreted as weak (0.10–0.39), moderate (0.40–0.69), or strong (≥ 0.7) (Schober et al. 2018).

Given the limitations of bivariate analyses in examining complex, multifactorial phenomena, such as back pain, network analysis was employed. This approach allowed for the simultaneous examination of relationships (edges) among multiple variables (nodes), offering a more nuanced understanding of data interactions (Borsboom et al. 2021; Hevey 2018). Specifically, network structures were estimated for all continuous variables and their subscales within each group (ABP, CBP, and pain‐free controls) to assess multivariate dependencies among variables (e.g., edge weights indicating the presence of absence of relationships between nodes; a positive or negative type of relationship; strength of the association between nodes). Thereby, the extended Bayesian information criterion graphical least absolute shrinkage and selection operator (EBICglasso) algorithm was used, combining regularisation with selecting the most relevant connections between variables, promoting a sparse and interpretable network structure (Isvoranu and Epskamp 2023). Finally, network structures were compared pairwise using the network comparison test, which employs resampling‐based permutation testing to assess differences between networks (van Borkulo et al. 2015; van Borkulo et al. 2023). Two key metrics were examined: (1) network invariance, which reflects the similarity in structural layout across networks; and (2) global strength, which measures the overall connectivity or intensity of associations within the networks (van Borkulo et al. 2023). Results were presented as *p*‐values, with statistical significance set at an alpha level of 0.05.

### 2.6 Sensitivity Analysis

To evaluate the robustness of our findings, we conducted complete case analyses, excluding individuals with missing data on any outcome measure or covariate. In addition, sensitivity analyses were performed treating well‐being and stressful life events as ordinal variables. Ordinal logistic regression models were used to examine group differences, adjusting for covariates. Benjamini‐Hochberg correction was applied for multiple comparisons (Benjamini and Hochberg 1995).

## 3 Results

### 3.1 Demographics

From the initial cohort of 502,185 individuals, 91,998 met our inclusion criteria (Figure 1). Among these, the majority were pain‐free controls (*n* = 80,484; 87%). After matching, the study ultimately included 22,864 participants, with 5716 in the ABP group, 5716 in the CBP group and 11,432 in the pain‐free control group. Descriptive statistics are presented in Table 1.

**TABLE 1:** Descriptive characteristics of the total study sample and separated by each group.

	Total study sample (*n* = 22,864)	Pain‐free controls (*n* = 11,432)	Acute back pain (*n* = 5716)	Chronic back pain (*n* = 5716)
	Mean (SD)	Mean (SD)	Mean (SD)	Mean (SD)
Age (years)	53.5 (8.1)	53.5 (8.1)	53.4 (8.1)	53.5 (8.0)
Sex (% male/female)	52/48	52/48	53/47	51/49
Body mass index (kg/m^2^)	26.2 (4.0)	26.0 (3.9)	26.3 (3.9)	26.4 (4.1)
Deprivation Index	−1.7 (2.9)	−1.7 (2.8)	−1.6 (2.9)	−1.7 (2.9)
Alcohol consumption *n* (%)				
Never	1164 (5.1)	602 (5.3)	285 (5.0)	277 (4.9)
Special occasions only	1761 (7.7)	902 (7.9)	435 (7.6)	424 (7.4)
One to three times a month	2309 (10.1)	1235 (10.8)	534 (9.3)	540 (9.5)
Once or twice a week	6186 (27.1)	3104 (27.2)	1537 (26.9)	1545 (27.0)
Three or four times a week	6375 (27.9)	3178 (27.8)	1595 (27.9)	1602 (28.0)
Daily or almost daily	5063 (22.1)	2409 (21.1)	1329 (23.3)	1325 (23.2)
Missing data	6 (0.03)	2 (0.02)	1 (0.02)	3 (0.05)
Smoking status *n* (%)				
Never	14,021 (61.3)	7318 (64.0)	3441 (60.2)	3262 (57.1)
Previous	6788 (29.9)	3201 (28.0)	1724 (30.2)	1863 (32.6)
Current	2008 (8.8)	896 (7.8)	540 (9.5)	572 (10.0)
Missing data	47 (0.2)	17 (0.2)	11 (0.2)	19 (0.3)

### 3.2 Well‐Being

**FIGURE 2:**
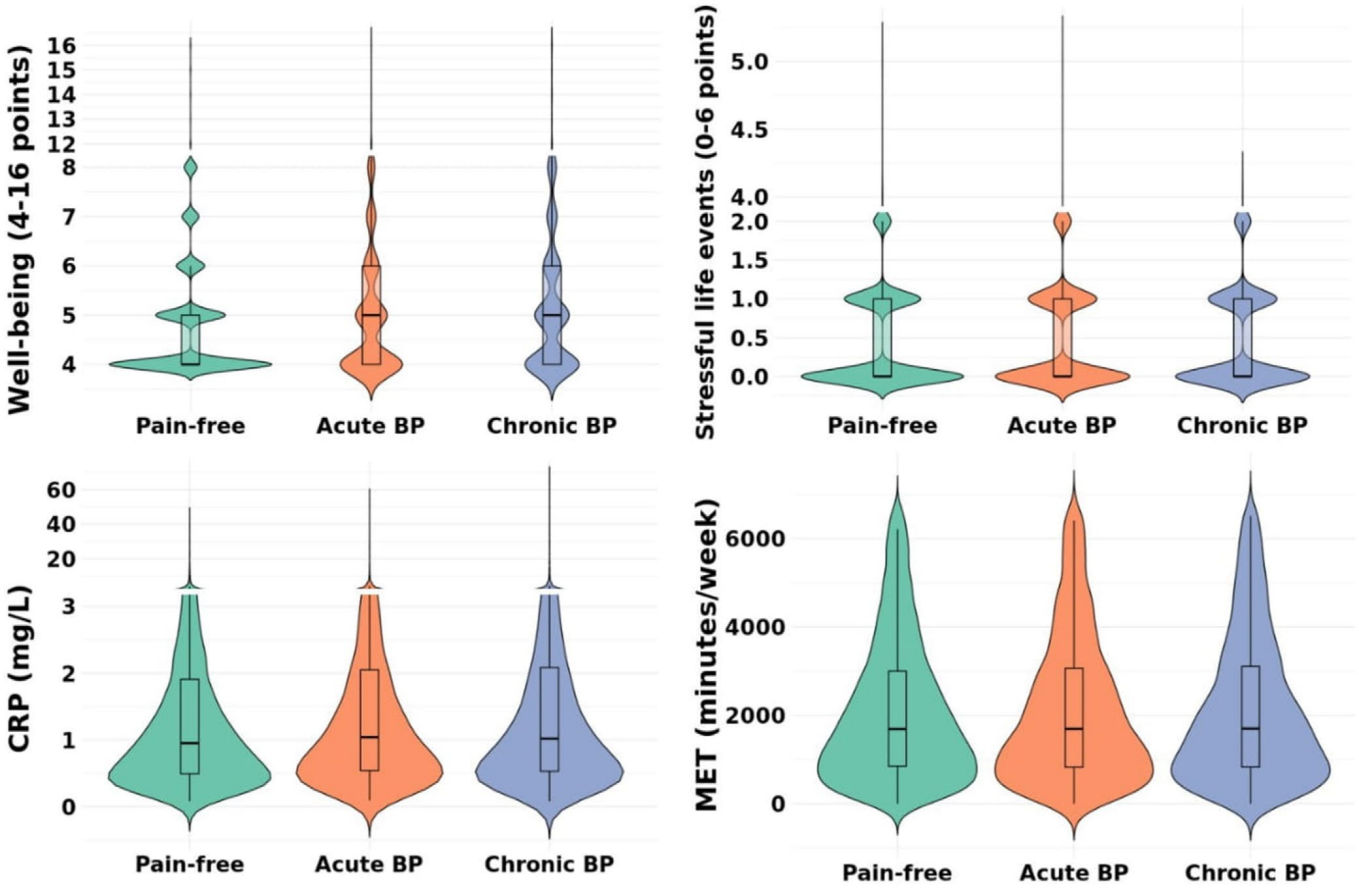
Violin plots of each outcome measure for pain‐free controls (green), acute back pain (orange), and chronic back pain (blue). The crossbars within each violin plot represent the median and interquartile range of the variable for each group.

Unadjusted results are presented in Table 2 and adjusted findings are presented in Table 3 and Figure 2. Well‐being was worse in both ABP [mean difference (MD: 4%; *d* = very small)] and CBP (8%; *d* = small) groups compared to the pain‐free control group. Individuals with CBP had worse well‐being scores than those with ABP (4%; *d* = very small).

**TABLE 2:** Unadjusted means (SE) and results of the analysis of variance (ANOVA) for all outcome measures.

	Pain‐free control		Acute back pain			Chronic back pain		
	Mean (SE)	*n*	Mean (SE), mean diff (95 CI)	*n*	*p*	Mean (SE), mean diff (95 CI)	*n*	*p*
Well‐being (4–16 points)	4.94 (0.01)	10,680	5.14 (0.02)	5206		5.34 (0.02)	5257	
Versus pain‐free			**0.21 (0.14, 0.27)**		**< 0.001**	**0.40 (0.34, 0.46)**		**< 0.001**
Versus acute back pain						**0.20 (0.12, 0.27)**		**< 0.001**
SLE (0–6 points)	0.47 (0.01)	11,305	0.50 (0.01)	5665		0.50 (0.01)	5662	
Versus pain‐free			**0.03 (0.00, 0.06)**		**0.018**	**0.03 (0.01, 0.06)**		**0.012**
Versus acute back pain						0.00 (−0.03, 0.03)		0.991
CRP (mg/L)^a^	1.76 (0.03)	10,702	1.90 (0.04)	5336		1.98 (0.05)	5347	
Versus pain‐free^b^			**4.06 (1.86, 6.26)**		**< 0.001**	**4.21 (2.01, 6.41)**		**< 0.001**
Versus acute back pain^b^						0.15 (−2.39, 2.69)		0.989
MET (minutes/week)	2096 (17.1)	8591	2124 (25.0)	4257		2128 (25.1)	4247	
Versus pain‐free			27.68 (−43.09, 98.45)		0.630	32.06 (−38.77, 102.88)		0.538
Versus acute back pain						4.37 (−77.51, 86.26)		0.991

*Note:* Data are reported as unadjusted mean (standard error), unadjusted mean differences, 95% confidence intervals (CI) and Cohen's *d* (*d*) unless specified. Bold indicates significant results (*p* < 0.05). *p*‐values were adjusted for multiple testing using the Tukey HSD method.

Abbreviations: CRP, C‐reactive protein; MET, metabolic equivalent of task; SLE, stressful life events.

^a^ Raw data is presented, however analyses and *p*‐values are based on log‐transformed data.

^b^ Mean percentage difference compared to reference category.

**TABLE 3:** Adjusted means (SE) and results of the analysis of covariance (ANCOVA) for all outcome measures.

	Pain‐free control	Acute back pain			Chronic back pain		
	Mean (SE)	Mean (SE), mean diff (95% CI)	*d*	*p*	Mean (SE), mean diff (95% CI)	*d*	*p*
Well‐being (4–16 points)	5.07 (0.02)	5.26 (0.03)			5.46 (0.02)		
Versus pain‐free		**0.20 (0.14, 0.25)**	**0.14**	**< 0.001**	**0.39 (0.34, 0.45)**	**0.26**	**< 0.001**
Versus acute back pain					**0.20 (0.14, 0.26)**	**0.12**	**< 0.001**
SLE (0–6 points)	0.50 (0.01)	0.53 (0.1)			0.53 (0.01)		
Versus pain‐free		**0.03 (0.01, 0.05)**	**0.04**	**0.041**	**0.03 (0.01, 0.05)**	**0.05**	**0.028**
Versus acute back pain					0.00 (−0.02, 0.03)	0.00	0.992
CRP (mg/L)^a^	1.99 (0.04)	2.07 (0.05)			2.13 (0.05)		
Versus pain‐free^b^		**2.28 (0.57, 3.99)**	**0.07**	**0.024**	1.98 (0.27, 3.69)	0.08	0.061
Versus acute back pain^b^					0.31 (−1.67, 2.28)	0.00	0.951
MET (minutes/week)	2083 (23.50)	2125 (29.20)			2130 (29.00)		
Versus pain‐free		42.39 (−16.66, 101.44)	0.02	0.337	46.77 (−12.37, 105.92)	0.02	0.268
Versus acute back pain					4.38 (−63.87, 72.64)	0.00	0.991

*Note:* Data are reported as adjusted mean (standard error), adjusted mean differences, 95% confidence intervals (CI) and Cohen's *d* (*d*) unless specified. Bold indicates significant results (*p* < 0.05). *p*‐values were adjusted for multiple testing using the Tukey HSD method. *d* from 0.2 to 0.49 were considered small, 0.5 to 0.79 were considered moderate, and ≥ 0.8 were considered large effects [6]. All data were adjusted for socioeconomic deprivation, body mass index, smoking status and alcohol consumption.

Abbreviations: CRP, C‐reactive protein; MET, metabolic equivalent of task; SLE, stressful life events.

^a^ Raw data is presented, however analyses and *p*‐values are based on log‐transformed data.

^b^ Mean percentage difference compared to reference category.

### 3.3 Stressful Life Events

Individuals with ABP had a higher number of stressful life events (6%; *d* = very small) (Table 3; Figure 2) compared to controls. The CBP group had a higher number of stressful life events compared to the pain‐free group (6%; *d* = very small). No difference in stressful life events was observed between ABP and CBP groups.

### 3.4 Inflammation

Higher levels of CRP were detected in individuals with ABP compared to pain‐free controls (2%; *d* = very small) (Table 3; Figure 2). There were no differences in CRP levels between the CBP group and pain‐free controls, nor between the CBP and ABP groups.

### 3.5 Physical Activity

No statistically significant differences were measured between groups for physical activity levels (Table 3; Figure 2).

### 3.6 Adjusted Partial Correlations

The adjusted partial correlation coefficients for each outcome measure across all groups and separated for each group are presented in Table 4. Across all groups, well‐being was the only weak positive correlation with stressful life events (*p* < 0.001). All other correlations were very weak: well‐being negatively correlated with MET (*p* < 0.001), while stressful life events showed a positive correlation with MET (*p* < 0.001). MET negatively correlated with CRP (*p* < 0.001). Similar results were observed in each separate group.

**TABLE 4:** Adjusted partial correlation coefficients for all outcome measures across all groups and for each separate group.

	Well‐being	SLE	CRP	MET
Across all groups				
Well‐being		0.13***	0.00	−0.06***
SLE	0.13***		−0.01	0.03***
CRP	0.00	−0.01		−0.03***
MET	−0.06***	0.03***	−0.03***	
Across pain‐free individuals				
Well‐being		0.13***	−0.01	−0.07***
SLE	0.13***		−0.00	0.03*
CRP	−0.01	−0.00		−0.02
MET	−0.07***	0.03*	−0.02	
Across acute back pain individuals				
Well‐being		0.15***	0.02	−0.07***
SLE	0.15***		0.00	0.03
CRP	0.02	0.00		−0.03
MET	−0.07***	0.03	−0.03	
Across chronic back pain individuals				
Well‐being		0.11***	0.01	−0.06***
SLE	0.11***		−0.03	0.02
CRP	0.01	−0.03		−0.05*
MET	−0.06***	0.02	−0.05*	

*Note:* Correlation coefficients were considered weak between 0.10 and 0.39, moderate between 0.40 and 0.69, and strong from 0.7 or higher [39]. All *p*‐values were adjusted according to the Benjamini‐Hochberg false discovery rate adjustment for multiplicity (Benjamini and Hochberg 1995). Partial correlations were adjusted for age, deprivation, smoking status, alcohol consumption, body mass index and the respective dependent variables not analysed.

Abbreviations: CRP, C‐reactive protein; MET, metabolic equivalent of task; SLE, stressful life events.

* *p* < 0.05.

*** *p* < 0.001.

### 3.7 Network Structures

**FIGURE 3:**
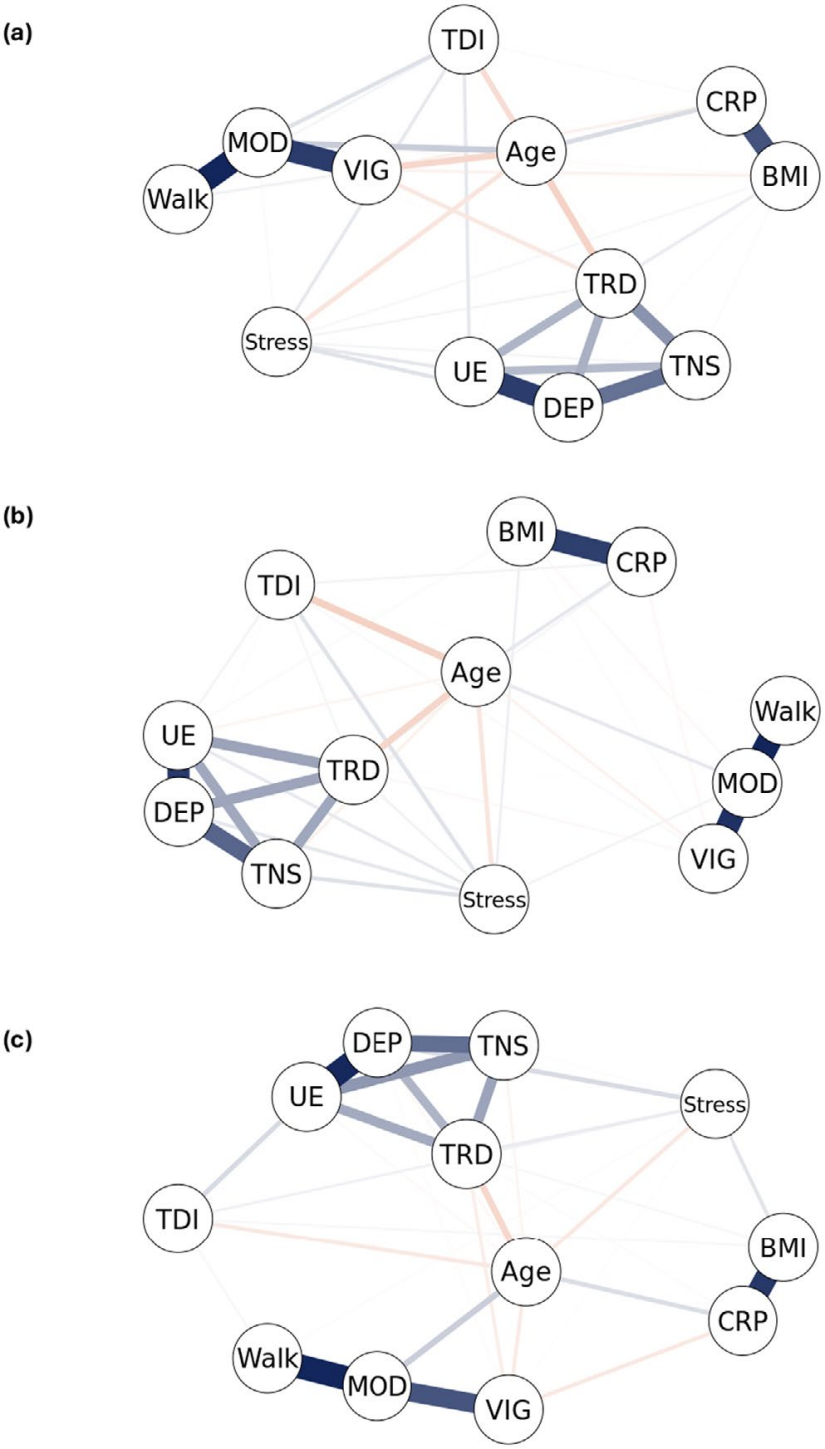
Network plots for pain‐free controls (a), acute back pain (b), and chronic back pain (c). Blue edges indicate a positive relationship, and orange edges a negative relationship between nodes. The thickness of the edges indicates the strength of a relationship. BMI, body mass index; CRP, C‐reactive protein; DEP, depressed; MOD, metabolic equivalent of moderate physical activity; Stress, stressful life events; TDI, Townsend deprivation index; TNS, tenseness; TRD, tiredness; UE, unenthusiasm; VIG, metabolic equivalent of vigorous physical activity; Walk, metabolic equivalent of walking.

Figure 3 depicts the network structures of all continuous variables and their subscales, separated by group. Across all three groups, marked positive relationships were observed within the subscales of well‐being (“Depressed,” “Unenthusiasm,” “Tiredness,” and “Tenseness”) as well as within the physical activity subscales (metabolic equivalent of vigorous physical activity [MET_vig], metabolic equivalent of moderate physical activity [MET_mod], and metabolic equivalent of walking [MET_walk]). Additionally, CRP demonstrated a strong positive correlation with BMI across all groups. While several relationships were identified between the subscales of well‐being and stressful life events, physical activity, and CRP, these associations were generally weak. The network comparison test revealed no statistically significant differences in network structure across the groups (Table 5). Similarly, there were no significant differences in global network strength between the groups.

**TABLE 5:** Results of the network comparison test between all groups based on network invariance and global strength.

Networks	Network invariance	Global strength
	*p*	*p*
Pain‐free vs. acute back pain	0.076	0.272
Pain‐free vs. chronic back pain	0.136	0.341
Acute back pain vs. chronic back pain	0.747	0.699

### 3.8 Sensitivity Analysis

In the complete case analyses, 7790 individuals were excluded due to missing data. Results were largely consistent with those previously reported (see Tables S1–S3; Figure S1). However, the *p*‐values for stressful life events in the comparisons between ABP versus pain‐free controls and CBP versus pain‐free controls were no longer statistically significant in the ANOVA and ANCOVA. The ordinal logistic regression results (Table S4) were generally consistent in direction and significance with the ANCOVA findings, particularly for well‐being outcomes. However, similar to the complete case analyses results, the odds of reporting more stressful life events were no longer significantly higher in individuals with ABP or CBP.

## 4 Discussion

Our study demonstrated differences in well‐being and CRP between ABP and CBP compared to pain‐free controls. Specifically, well‐being was worse in both ABP and CBP compared to controls, with CBP reporting the poorest well‐being. Back pain groups experienced more stressful life events than controls, but this finding was not robust to sensitivity analyses and should therefore be interpreted with caution. Furthermore, ABP had a higher CRP than pain‐free controls. No differences were detected in physical activity between groups. Partial correlations showed mostly very small relationships among variables, with the only weak correlation observed between well‐being and stressful life events. Network analysis revealed strong intra‐group correlations within well‐being and physical activity subscales, but weak relationships between well‐being, stressful life events, physical activity, and CRP, with no differences in structure or global strength across groups.

### 4.1 Well‐Being, Stressful Life Events and Back Pain

Consistent with a previous study (Tagliaferri et al. 2022), we observed worse well‐being in ABP, with poorer well‐being in CBP compared to controls. However, our analysis revealed smaller mean differences than those reported in the previous study for ABP versus pain‐free (MD [95% CI] = 0.2 [0.1, 0.3] vs. 0.3 [0.2, 0.4]), CBP vs. pain‐free (0.4 [0.3, 0.5] vs. 0.5 [0.4, 0.7]), and CBP vs. ABP (0.2 [0.1, 0.3] vs. 0.3 [0.1, 0.4]). These discrepancies may be due to stricter inclusion criteria and additional covariate adjustments in our study. It is important to consider that well‐being was assessed using the PHQ‐4, an ultra‐brief self‐reported questionnaire comprising two items for both depressive and anxiety symptoms. While the PHQ‐4 has acceptable psychometric properties, the brevity limits capturing the full spectrum of depressive and anxiety symptoms and does not address back pain‐specific domains (Kroenke et al. 2009). The use of a more comprehensive, back pain‐specific instrument could yield more nuanced results. Furthermore, no minimal clinically important difference (MCID) exists for the PHQ‐4.

Stressful life events were also more prevalent in both ABP and CBP compared to pain‐free controls; however, effect sizes were very small, and results were not supported by sensitivity analysis. Notably, the number of stressful life events only relates to the past 2 years and does not capture the subjective perception of stress, which can vary widely among individuals (Wu et al. 2013). Future research could incorporate perceived stress measures to clarify the relationship between stress and back pain.

### 4.2 Inflammation and Back Pain

We observed that individuals with ABP had higher CRP compared to pain‐free controls. However, the mean difference did not reach the MCID of 10.0 mg/L for healthy populations (Morley and Kushner 1982). Moreover, no differences were observed between CBP and either ABP or pain‐free controls. Importantly, CRP levels across all groups remained within the normal range of < 5 mg/L as defined for the general population (Gebhardt et al. 2006). Consequently, CRP may have limited clinical utility when comparing ABP and CBP. However, given CRP is an acute phase reactant and known to be higher in the early course of injury and decline, such as within the first 3 weeks of back pain onset (Gebhardt et al. 2006), exploring potential utility during this period warrants consideration. Identifying heightened CRP in a specific phase of ABP could provide valuable insights into the recovery trajectory.

Previous research on CRP in back pain has been inconclusive. Although systematic reviews generally reported elevated CRP, most included studies did not differentiate between ABP and CBP (Lim et al. 2020; Pinto et al. 2023). Among the three small studies that made this distinction, two support our findings. First, Klyne et al. (2017) documented higher CRP in 99 ABP individuals (median [interquartile range] = 1.6 [0.4–3.2] mg/L) compared to 55 healthy young adults (1.0 [0.4–1.8] mg/L). Similarly, Hao‐Wei et al. (2021) reported higher CRP in 60 ABP (mean [SE] = 5.99 ± 2.56 ng/mL) compared to 78 CBP (5.14 ± 2.36 ng/mL) and to 60 healthy controls (4.43 ± 0.52 ng/mL) of similar age to UK Biobank participants. In contrast, Gebhardt et al. (2006) found no differences in CRP among 31 ABP, 41 CBP and 1572 pain‐free controls, highlighting the need for larger studies that distinguish between ABP and CBP.

### 4.3 Physical Activity and Back Pain

Physical activity is often lower in CBP compared to healthy individuals (Lin et al. 2011); however, we found no differences between ABP, CBP, and controls. This result aligns with some reviews (Griffin et al. 2012; Hendrick et al. 2011), but contrasts with others, which found that CBP individuals are likely to have lower levels of physical activity (Lin et al. 2011; Shiri and Falah‐Hassani 2017). The results should be interpreted in the context that the IPAQ is a self‐reported measure with acceptable validity (Craig et al. 2003), and only evaluates total physical activity levels. Future studies could use objective measures and assess various dimensions (i.e., type, frequency, duration and intensity) and domains of physical activity (i.e., leisure time, organised sport, household, commuting and work) to provide a comprehensive understanding of how different forms of activity impact back pain (Shiri and Falah‐Hassani 2017).

### 4.4 Correlations and Network Analysis

Very small but consistent bivariate correlations were observed between well‐being, stressful life events, CRP, and physical activity across groups, aligning with a UK Biobank study that examined other psychosocial variables (e.g., depression, loneliness) in back pain (Tagliaferri et al. 2022). Network analysis offered additional insights into multivariate dependencies among variables, revealing strong positive associations within well‐being and physical activity subscales, as well as between CRP and BMI. While substantial evidence links poor well‐being with elevated inflammation (Kiecolt‐Glaser et al. 2010) and higher physical activity with both higher well‐being (Saqib et al. 2020) and anti‐inflammatory effects (Kim et al. 2008), we found only weak relationships among well‐being, stressful life events, CRP, and physical activity. This may be due to the exclusive focus on CRP rather than a broader range of inflammatory biomarkers (e.g., TNF‐α, IL‐1β, IL‐6), limiting the ability to capture the full spectrum of inflammatory processes potentially involved in back pain. Moreover, limitations in the instruments used to assess well‐being and physical activity (e.g., not distinguished for physical activity during leisure time, organised sport, household, commuting and work) may have influenced the findings.

Network structures were similar across groups, indicating that interactions between well‐being, CRP, and physical activity remain consistent across ABP, CBP, and pain‐free controls. Consequently, when considering only these variables, there is no evidence of neuroimmune involvement in ABP or pain chronification. Although no previous studies have employed network analysis for ABP and CBP, network analyses in other chronic pain cohorts showed consistently strong associations within psychological variables (e.g., between depressive and anxious symptoms) (Akerblom et al. 2021; Penedo et al. 2020; Thompson et al. 2019), aligning with our findings. Previous studies included a wider range of psychological (e.g., catastrophising, fear‐avoidance) and pain‐related variables (e.g., pain intensity, interference), yielding more comprehensive network structures between mental health and pain. However, variables on inflammation or physical activity were not included. Future research could incorporate additional variables for a more nuanced understanding of back pain.

### 4.5 Strengths and Limitations

Strengths of the current study included using the largest ABP and CBP sample examining CRP to date, strict inclusion criteria that controlled for known covariates, pairwise matching to enhance comparability, and network analysis that provided a novel multivariate approach. However, there are limitations. First, the cross‐sectional design precluded causal inferences. Second, the network analysis only included a limited number of variables and future analyses could benefit from including further biopsychosocial markers. Third, self‐reported measures increased the likelihood of recall bias. Fourth, back pain classification lacked detail on pain duration and intensity. Fifth, CRP was our only measure of inflammation and may not reflect other markers, such as TNF‐α, IL‐1β and IL‐6, commonly associated with ABP and CBP (Morris et al. 2020). Lastly, alternative approaches such as propensity score matching could leverage the full sample and allow for simultaneous adjustment across multiple covariates, potentially offering more robust control of confounding variables.

## 5 Conclusions

This study found differences across ABP, CBP, and pain‐free individuals in well‐being and CRP, suggesting that both variables may be influenced by the temporality of back pain. In addition, consistent network structures across groups indicate no neuroimmune involvement based on the variables examined. Overall, prospective studies examining further variables are needed to better understand biopsychosocial drivers of back pain.

## Author Contributions

Conceptualisation: All. Data curation: R.G. Formal Analysis: R.G. Funding acquisition: R.G., L.C.M. Investigation: R.G. Methodology: All. Project administration: All. Resources: R.G. Software: R.G. Supervision: L.C.M., J.L.T., P.J.O. Validation: P.J.O. Visualisation: R.G. Writing – original draft: R.G. Writing – review and editing: All.

## Conflicts of Interest

The authors declare no conflicts of interest.

## Supporting information


Data S1.

## Data Availability

Publicly available UK Biobank data were analysed in this study. Researchers can access data sets through open registration (see https://www.ukbiobank.ac.uk). The full statistical code is provided on a public data repository (https://github.com/RomyGollan/UKBiobank_back_pain.git) to independently reproduce analyses.
