# Supplementary material for: Well‐Being, Inflammation, and Physical Activity in Acute and Chronic Back Pain: A Cross‐Sectional Analysis of 22,864 UK Biobank Participants
Source: Eur J Pain. 2025 Jul 20;29(7):e70079. doi: 10.1002/ejp.70079 (PMC12277551; doi:10.1002/ejp.70079)
Supplement: Supplementary file 1 — Data S1. [file EJP-29-0-s001.docx]

**Supplement**

*Tables*

Table S1. Unadjusted means (SE) and results of the analysis of variance (ANOVA) for all outcome measures, after excluding individuals with missing data.

|  | Pain-free control | | Acute back pain | | | Chronic back pain | | |
| --- | --- | --- | --- | --- | --- | --- | --- | --- |
|  | Mean (SE)  Mean diff (95 CI) | n | Mean (SE)  Mean diff (95 CI) | n | *p* | Mean (SE)  Mean diff (95 CI) | n | *p* |
| Well-being (4-16 points) | 4.93 (0.02) | 7,625 | 5.11 (0.03) | 3,722 |  | 5.31 (0.03) | 3,727 |  |
| vs Pain-free |  |  | **0.19 (0.11, 0.26)** |  | **<0.001** | **0.38 (0.31, 0.46)** |  | **<0.001** |
| vs Acute back pain |  |  |  |  |  | **0.20 (0.12, 0.29)** |  | **<0.001** |
| SLE (0-6 points) | 0.47 (0.01) | 7,625 | 0.48 (0.01) | 3,722 |  | 0.50 (0.01) | 3,727 |  |
| vs Pain-free |  |  | 0.01 (-0.02, 0.04) |  | 0.756 | 0.02 (-0.02, 0.06) |  | 0.414 |
| vs Acute back pain |  |  |  |  |  | 0.00 (-0.03, 0.03) |  | 0.991 |
| CRP (mg/L)^🞦^ | 1.75 (0.03) | 7,625 | 1.88 (0.05) | 3,722 |  | 1.95 (0.06) | 3,727 |  |
| vs Pain-free^⬥^ |  |  | **4.22 (1.59, 6.85)** |  | **<0.001** | **3.88 (1.25, 6.51)** |  | **0.002** |
| vs Acute back pain^⬥^ |  |  |  |  |  | -0.34 (-3.39, 2.71) |  | 0.964 |
| MET (minutes/week) | 2090 (18.2) | 7,625 | 2129 (26.7) | 3,722 |  | 2135 (26.8) | 3,727 |  |
| vs Pain-free |  |  | 39.85 (-35.57, 115.28) |  | 0.430 | 45.25 (-30.14, 120.64) |  | 0.337 |
| vs Acute back pain |  |  |  |  |  | 5.40 (-82.01, 92.81) |  | 0.989 |

Data are reported as adjusted mean (standard error), adjusted mean differences, 95% confidence intervals (CI) and Cohen’s *d* (*d*) unless specified. ^🞦^raw data is presented, however analyses and p-values are based on log-transformed data. ^⬥^mean percentage difference compared to reference category. Bold indicates significant results (*p* < 0.05). P-values were adjusted for multiple testing using the Tukey HSD method. SLE , Stressful life events; CRP, C-reactive protein; MET, Metabolic equivalent of task.

Table S2. Adjusted means (SE) and results of the analysis of covariance (ANCOVA) for all outcome measures, after excluding individuals with missing data.

|  | Pain-free control | Acute back pain | | | Chronic back pain | | |
| --- | --- | --- | --- | --- | --- | --- | --- |
|  | Mean (SE) | Mean (SE),  mean diff (95% CI) | *d* | *p* | Mean (SE),  mean diff (95% CI) | *d* | *p* |
| Well-being (4-16 points) | 5.05 (0.02) | 5.23 (0.03) |  |  | 5.43 (0.03) |  |  |
| vs Pain-free |  | **0.18 (0.12, 0.24)** | **0.13** | **<0.001** | **0.38 (0.32, 0.44)** | **0.25** | **<0.001** |
| vs Acute back pain |  |  |  |  | **0.20 (0.13, 0.27)** | **0.12** | **<0.001** |
| SLE (0-6 points) | 0.50 (0.01) | 0.50 (0.1) |  |  | 0.52 (0.01) |  |  |
| vs Pain-free |  | 0.01 (-0.02, 0.03) | 0.01 | 0.874 | 0.03 (-0.00, 0.05) | 0.04 | 0.160 |
| vs Acute back pain |  |  |  |  | 0.02 (-0.01, 0.05) | 0.03 | 0.482 |
| CRP (mg/L)^🞦^ | 1.75 (0.03) | 1.88 (0.05) |  |  | 1.95 (0.06) |  |  |
| vs Pain-free^⬥^ |  | **2.62 (0.58, 4.67)** | **0.08** | **0.032** | 1.55 (0.49, 3.60) | 0.07 | 0.297 |
| vs Acute back pain^⬥^ |  |  |  |  | -1.07 (-3.44, 1.30) | 0.01 | 0.649 |
| MET (minutes/week) | 2074 (25.10) | 2125 (31.20) |  |  | 2133 (30.90) |  |  |
| vs Pain-free |  | 51.08 (-11.66, 113.82) | 0.02 | 0.247 | 59.61 (-3.18, 122.41) | 0.03 | 0.150 |
| vs Acute back pain |  |  |  |  | 8.53 (-64.13, 81.19) | 0.00 | 0.971 |

Data are reported as adjusted mean (standard error), adjusted mean differences, 95% confidence intervals (CI) and Cohen’s *d* (*d*) unless specified. ^🞦^raw data is presented, however analyses and p-values are based on log-transformed data. ^⬥^mean percentage difference compared to reference category. Bold indicates significant results (*p* < 0.05). *P*-values were adjusted for multiple testing using the Tukey HSD method. *d* from 0.2 to 0.49 were considered small, 0.5 to 0.79 were considered moderate, and ≥ 0.8 were considered large effects [6]. All data were adjusted for socioeconomic deprivation, body mass index, smoking status and alcohol consumption. SLE, stressful life events; CRP, C-reactive protein; MET, Metabolic equivalent of task.

Table S3. Results of the network comparison test between all groups based on network invariance and global strength, after excluding individuals with missing data.

| Networks | Network invariance | Global strength |
| --- | --- | --- |
|  | *p* | *p* |
| Pain-free vs Acute back pain | 0.064 | 0.200 |
| Pain-free vs Chronic back pain | 0.104 | 0.054 |
| Acute back pain vs Chronic back pain | 0.786 | 0.581 |

Table S4. Results of the ordinal logistic regression for well-being and stressful life events.

| Predictor | Well-being |  | SLE |  |
| --- | --- | --- | --- | --- |
|  | OR (95% CI) | *p* | OR (95% CI) | *p* |
| Group (ref = Pain-free control) | | | | |
| Acute back pain | **1.33 (1.21, 1.47)** | **<0.001** | 1.11 (0.99, 1.25) | 0.138 |
| Chronic back pain | **1.66 (1.51, 1.81)** | **<0.001** | 1.09 (0.97, 1.22) | 0.235 |
| Deprivation Index | **1.08 (1.07, 1.10)** | **<0.001** | **1.06 (1.04, 1.07)** | **<0.001** |
| BMI | **1.01 (1.00, 1.02)** | **0.029** | **1.03 (1.02, 1.04)** | **<0.001** |
| Smoking status (ref = Never) | | | | |
| Current | **1.62 (1.43, 1.83)** | **<0.001** | **1.37 (1.17, 1.60)** | **<0.001** |
| Previous | **1.14 (1.04, 1.24)** | **0.006** | 1.04 (0.94, 1.16) | 0.552 |
| Alcohol consumption (ref = Never) | | | | |
| Daily or almost daily | **0.72 (0.60, 0.86)** | **<0.001** | 0.90 (0.71, 1.13) | 0.483 |
| 3–4 times/week | **0.65 (0.55, 0.78)** | **<0.001** | 0.94 (0.75, 1.17) | 0.666 |
| Once or twice/week | **0.79 (0.66, 0.94)** | **0.010** | 0.84 (0.67, 1.05) | 0.222 |
| 1–3 times/month | **0.79 (0.65, 0.96)** | **0.022** | 0.98 (0.76, 1.25) | 0.856 |
| Special occasions | 1.01 (0.82, 1.23) | 0.945 | 1.02 (0.79, 1.32) | 0.856 |

OR, odds ratio; Ref, reference; SLE, stressful life events. The Benjamini-Hochberg procedure was applied to control for false discovery rate, adjusting the *p*-values derived from multiple hypothesis testing (Benjamini & Hochberg, 1995).

*Figures*


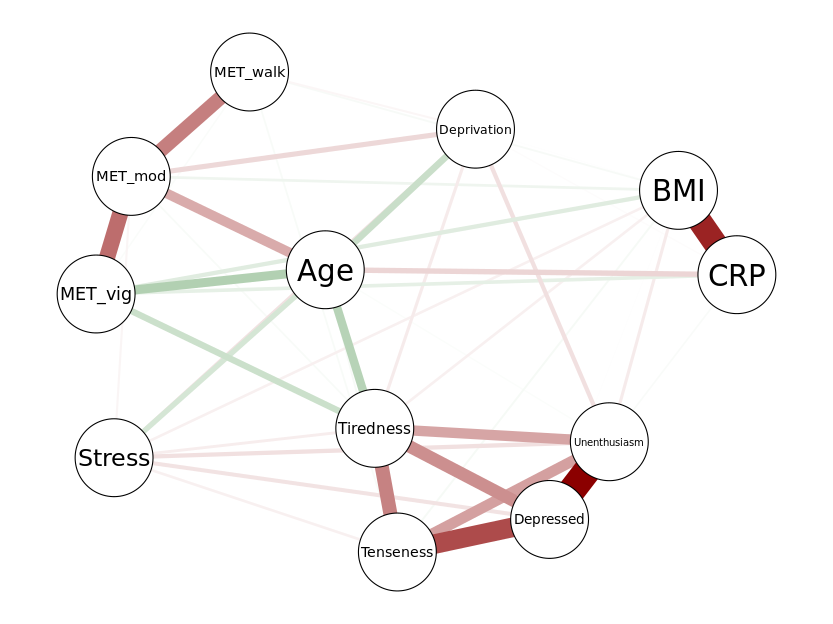

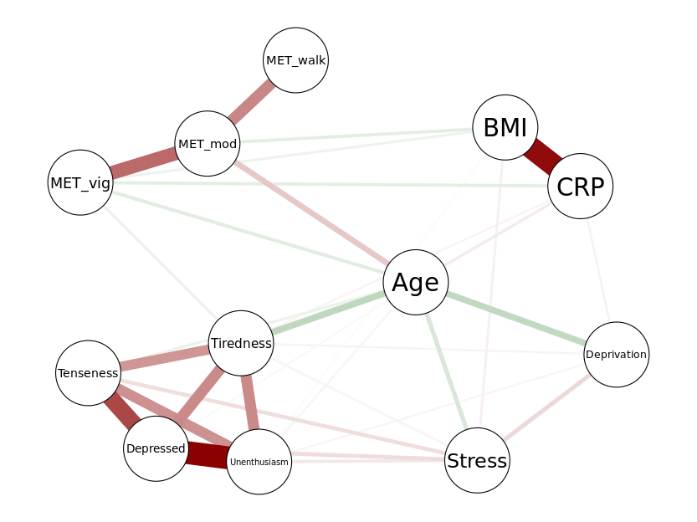

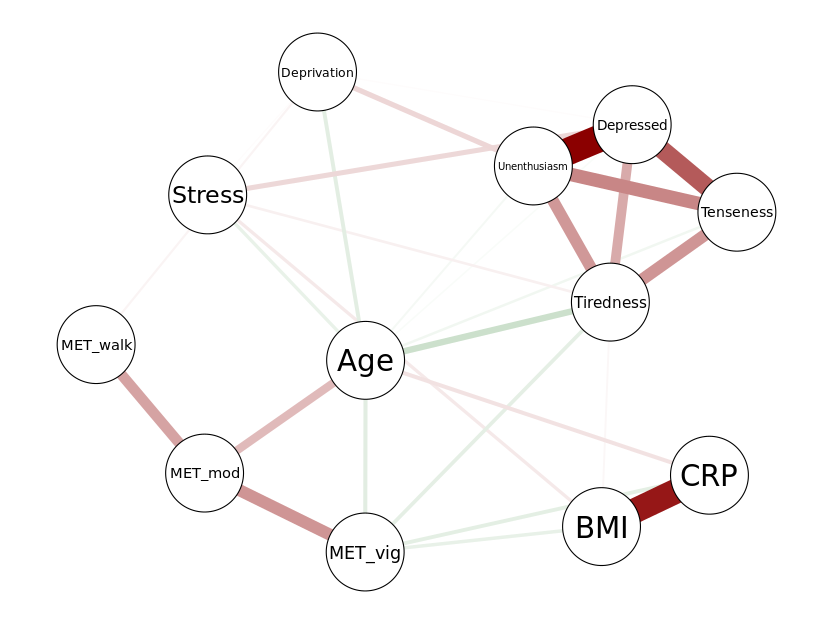


**Pain-free**

**Acute BP**

**Chronic BP**

Figure S1. Network plots for pain-free controls, acute back pain, and chronic back pain after excluding individuals with missing data. Red edges indicate a positive relationship and green edges a negative relationship between nodes. The thickness of the edges indicates the strength of a relationship. BMI, body mass index; CRP, C-reactive protein. Deprivation = Townsend deprivation index; Stress = Stressful life events; MET_walk = metabolic equivalent of walking; MET_mod = metabolic equivalent of moderate physical activity; MET_vig = metabolic equivalent of vigorous physical activity.
